# Biostimulation potential of biochar for remediating the crude oil contaminated soil and plant growth

**DOI:** 10.1016/j.sjbs.2021.03.044

**Published:** 2021-03-22

**Authors:** Maimona Saeed, Noshin Ilyas, Krish Jayachandran, Shagufta Gaffar, Muhammad Arshad, Muhammad Sheeraz Ahmad, Fatima Bibi, Kaouthar Jeddi, Kamel Hessini

**Affiliations:** a Department of Botany, PMAS-Arid Agriculture University Rawalpindi, 46300 Rawalpindi, Pakistan; b Earth and Environment Department, Florida International University, USA; c Department of Biochemistry, PMAS-Arid Agriculture University Rawalpindi, 46300 Rawalpindi, Pakistan; d Laboratory of Plant Biodiversity and Dynamic of Ecosystems in Arid Area, Faculty of Sciences of Sfax, B.P. 1171, Sfax 3000, Tunisia; e Department of Biology, College of Sciences, Taif University, P.O. Box 11099, Taif 21944, Saudi Arabia

**Keywords:** Biochar, Crude oil, Maize, Biostimulation

## Abstract

Crude oil contamination is a serious environmental threat to soil and plants growing in it. Biochar has the potential of biostimulation for remediation of crude oil-contaminated soil. Therefore, the current research was designed to analyze the bio-stimulatory impact of biochar for remediating the crude oil contaminated soil (10%, and 15%), and growth of maize under glasshouse conditions. Biochar was produced by pyrolysis of Australian pines at 350 °C. Soil incubations were done for 20 days. The results of soil analysis showed that the crude oil degradation efficiency of biochar was 34%. The soil enzymatic activities had shown 38.5% increase in fluorescein diacetate (FDA) hydrolysis and 55.6% increase in dehydrogenase activity in soil incubated with biochar in comparison to control. The soil microbial diversity was improved to 41% in biochar treated soil with respect to untreated one, while microbial respiration rate had shown a 33.67% increase in soil incubated with biochar with respect to control under oil stress. Gas Chromatography Mass spectrometry (GC-MS) analysis had shown the high content of low molecular weight hydrocarbons (C_9_-C_13_) in the soil incubated with biochar in comparison to untreated soil. Biochar showed a significant increase in fresh and dry biomass (25%, 14.61%), leaf area (10%), total chlorophyll (11%), water potential (21.6%), osmotic potential (21%), and membrane stability index (12.7%). Moreover, biochar treatment showed a higher increase in the contents of proline (29%), total amino acids (18%), soluble sugars (30.4%), and antioxidant enzymes like superoxide dismutase (16.5%), catalase (11%), and peroxidase (12%). Overall, the results of the present study suggest the bio-stimulating potential of biochar for degradation of hydrocarbons in crude oil contaminated soil and their growth-stimulating effects on maize.

## 1 Introduction

Soil contamination with petroleum hydrocarbons is considered to be an emerging issue currently. The main components of petroleum hydrocarbons are carbon, hydrogen, oxygen and in some cases also contain nitrogen and sulfur as well. Straight chain and ring-shaped hydrocarbons, colloid, and asphaltene are the basic components of total petroleum hydrocarbons (TPH) which are deadly poisonous and can not be broken down speedily by soil indigenous flora (Zhang et al., 2019). Moreover, weathering can stimulate the blockage of soil pores, resulting in enduring complications of soil mortality and reduction of biota bioactivity and degradability to contaminants (Lominchar et al., 2018, Rahbari-Sisakht et al., 2017). Although many approaches for remediation of petroleum contaminated soil have been recommended, still there is a need for effective and eco-friendly techniques for hydrocarbon removal. Many researchers suggested bioremediation, which can be practically applied as an economical and ecologically reliable technique (Wu et al., 2016). Recently, biostimulation and bioaugmentation are two actively used approaches. Biostimulation comprises actions that enhance the native microbial flora of oil-contaminated soil and bioaugmentation is the addition of microbes for the degradation of pollutants. Few studies described the efficiency of biostimulation and less supplementary advantage from bioaugmentation (Rajapaksha et al., 2016). Some researchers explained the combined effect of biostimulation and bioaugmentation. The combination of both techniques can stimulate the activities of microbes and improve soil properties. As the presence of hydrocarbons inhibits the activities of indigenous microbes due to toxicity of hydrocarbons, nutrient deficiency, and competition of microbes.

The bacterial activity can be enhanced by adding the stimulant in soil that can provide better conditions for bacterial activities and helps to tolerate unfavorable conditions. Various recent researches have explained that the addition of agricultural wastes like peanut shells, rice straws, and biochar can be used as a stimulant for microbial activities (Xue et al., 2019). These materials are capable of carrying oxygen, retaining water, and enhancing enzymatic activities (Shi et al., 2018).

Biochar, a carbon-rich residue formed by the pyrolysis of organic matter. Biochar has high adsorption ability, better stability, and the maximum ability for nutrient absorption as compared to other agricultural wastes (Liu et al., 2016; Ali et al., 2019, Mansoor et al., 2021). Biochar has a major positive effect on the soil, as it reduces the release of CO_2_, improves soil porosity and pH (He et al., 2016). Such characteristics of biochar help in the establishment and stimulation of indigenous microorganisms (Wang et al., 2015). Many lignin and cellulose biochars can be used for the growth and division of microbes. Along with abiotic factors, biochar can stimulate the activities of enzymes like dehydrogenase, polyphenol oxidase, and fluorescein diacetate (FDA) hydrolase and cycling of elements in the soil (Zhang et al., 2019). Biochar can absorb various organic and inorganic substances. Many studies reported that this can be an incentive as sorption decreases the liability and concentration of soil toxicants, resulting in the reduction of plant toxicity (Lu et al., 2014). Furthermore, the more surface area, soil porosity, and presence of functional groups of biochar may stimulate the adsorption capacity of biochar and the impact of contaminants (Denyes et al., 2016). Various types of biomass are used for the preparation of biochar. However, the use of pine needles for the production of biochar has certain advantages due to some beneficial characteristics of pine needles. Pine needles have a high volatile matter with a low moisture content which is favorable for pyrolysis. Pine needles have less content of sulphur and nitrogen so the emission of toxic gases like oxides of nitrogen and sulphur is very low (Varma and Mondal, 2017)

Various reports in the literature have documented the effect of bacteria on the remediation of polluted soil (Li et al., 2016). Some studies have explained the potential of biochar for hydrocarbons degradation by stimulating the activities of indigenous microbes. Biochar impacts the activities of indigenous microbes to stimulate the degradation of hydrocarbons (Kong et al., 2018). Consequently, biochar and decaying petroleum bacteria have been studied in various combinations including immobilization, bacteria, and biochar alone, free bacteria- biochar. However, the bio stimulating effect of biochar on soil indigenous bacteria for hydrocarbon degradation and the use of polluted soil for plant growth has been documented in few published reports. Therefore, the present research was designed to determine the potential of biochar as a bio stimulating tool to degrade hydrocarbons for remediation of oil-contaminated soil. The efficacy of biochar was confirmed by soil characterization, enzymatic activity, and microbial activity. Plant growth promoting effect was determined by growing maize with biochar incubation at treated soil.

## 2 Material and methods

### 2.1 Sample collection and biochar preparation

Australian Pines were collected from local farms of South Florida Homestead. Biochar was prepared by pyrolyzing Australian pine at 350 °C temperature in a closed container at USDA South Carolina. After pyrolization, the biochar was cooled, grounded, and passed through a 2 mm sieve to have a small particle size. Moisture content, volatile matter, ash content, and fixed carbon were studied in triplicates by the proximate analytical process for wood by ASTM for wood charcoals (ASTM D1762-84, reapproved 2007). The samples were sent to GALBRAITH LABORATORIES, INC. Knoxville, TN, for elemental analysis. Elemental analysis was done by using atomic absorption spectrophotometer (Enders et al., 2012).

### 2.2 Potential of biochar for growth attributes of maize experiment

A pot experiment was carried out in the organic garden of Florida International University USA, from April 2019 to June 2019. The relative humidity differed between 62 and 81% and day length from 12 to 13 h. Earthen pots with 10 Kg capacity of the soil were selected and filled with a 1:3 ratio of sand and soil. 1% biochar w/w was incubated in soil 20 days before sowing. The soil was contaminated at a 10% and 15% level by adding diesel oil v/w before sowing. Maize seeds were obtained from the University of Florida. The treatments were as follows: (1) To = Control without oil and biochar (2) T1 = Soil with 10% oil contamination (3) T2 = Soil with 15% oil contamination (4) T3 = Soil with 1% biochar T4 = Soil with 10% oil contamination and biochar, T5 = soil with 15% oil contamination and biochar. In each pot, 3 seeds were allowed to germinate. Plants were thinned out after 1 week of seed germination to produce one plant per pot. In a complete randomization method, the experiment was structured. Pots were maintained during the experiment in a well-watered state. Soil and plant samples were obtained for examination after 40 days of the experiment.

### 2.3 Soil analysis

Soil samples were studied before and after the remediation. The biochar and soil pH was determined with pH meter and electrical conductivity (EC) was measured by EC meter (Radojevic and Bashkin, 2007) and the organic matter (OM) was estimated with the volumetric potassium dichromate process (Giovannini et al., 1985). Soil moisture content was determined by following the procedure of Priha and Smolander (1999).

### 2.4 Determination of hydrocarbons content of soil sample

The soil samples were freeze-dried at the end of the experiment. Total petroleum hydrocarbons (TPHs) of soil were determined by the gravimetric method. Briefly, 5 g soil was mixed in 30 mL of methylene chloride and the extract was prepared with ultrasonication for 15mins by wrapping filter paper. Then, this wrapping filter paper was placed in a Soxhlet extractor for 12 h at 54 °C water bath and concentrated in a rotary evaporator. The final volume was made up to 50 mL. The concentration of remaining total petroleum hydrocarbons (TPHs) was gravimetrically computed. A gas chromatograph-mass spectrometer calculated the content of n-alkanes (GC-MS, model 7890-5975C, Agilent Technologies, USA). The elimination of TPHs (percentage) was calculated using the following formula:

(1) TPHs removal (%) = [(*w*_0_ − *w_t_*)/*w*_0_] × 100

where w_0_ is the initial concentration of soil TPHs (g kg^−1^), w_t_ stands for the concentration of residual TPHs at time t (g kg^−1^), and t is the remediation time (day).

### 2.5 Soil enzymatic analysis

An ultraviolet spectrophotometer (PERSEE TU-1901) was used to evaluate dehydrogenase, fluorescein diacetate (FDA) hydrolysis activities. The activity of dehydrogenase was assessed by incubating 1 g soil with 1 mL of triphenyltetrazolium chloride (TTC) at 30 °C for 6 h. At 485 nm, the existence of triphenyl formazan (TPF) was spectrophotometrically analyzed. FDA hydrolysis activity was measured by taking 1 g soil and added 10 mL of 100 mmol/L potassium phosphate buffer (pH 7.0) and 0.2 mL of 1 mg m/L FDA solution. The whole content was placed for 1 h at 30 °C and extraction was done by using chloroform/methanol in the ratio of 1:1 (V/V). The presence of fluorescein was analyzed spectrophotometrically at 490 nm. All samples were carried out in triplicate (Rostami and Rostami, 2019, Safari et al., 2018).

### 2.6 Soil microbial respiration rate and bacteria diversity analysis

The soil microbial respiration rate was calculated by the alkali absorption procedure (Liu et al., 2010a, Liu et al., 2010b). The amount of CO_2_ emitted from the soil microbes was measured by the use of HCl. To study the hydrocarbon-degrading bacteria, the plate counting protocol was followed, described as 5 g of soil sample was mixed and mixed well in 100 mL of autoclaved distilled water. The suspension was then diluted serially and 0.2 mL of solution was spread uniformly over the medium surface. The petroleum degrading bacteria were incubated as a carbon source with a mineral salt medium having 50 mg/L crude oil. The colonies appearing on crude oil containing MSM were counted as petroleum degrading bacteria after 5 days (Meng and Chi, 2017).

### 2.7 Plant analysis

After 4 weeks, the plants were uprooted, washed with distilled water. Plant fresh biomass was measured. Afterward, plants were oven-dried at 65 °C for 72 h and dry biomass was determined. A leaf area meter (AM300 leaf area meter) was used to determine the leaf area of all samples for each treatment. Before harvesting plants, samples were collected for various physiological, biochemical parameters, and antioxidant enzyme assays.

#### 2.7.1 Physiological parameters

A Scholander pressure chamber (670 Model, USA) was used for the determination of leaf water potential (Scholander et al., 1965). Arnon's (1949) method was used for estimating the chlorophyll content of leaf samples. Leaves were weighed and then ground in a clean pestle and mortar. Later on, each sample was mixed in 5 mL of 80% acetone. After centrifugation, the supernatant was separated. The absorbance of the extract was measured at various wavelengths i.e., 663 nm and 645 nm with a spectrophotometer. The values of chlorophyll *a*, b, total chlorophyll were calculated by the following formula.

Chlorophyll *a* (µg/mL) = 12.21 (A_663_) − 2.81 (A_645_)

Chlorophyll *b* (µg/mL) = 20.13 (A_645_) – 5.03 (A_663_)

Total chlorophyll (µg/mL) = 20.2 (A_645_) + 8.02 (A_663_)

The osmotic potential was determined by opting for the procedure of Capell and Doerffling (1993). Leaves from each treatment were placed in a 3 mL plastic syringe and preserved at −20 °C freezer. After a few days, when the leaves became frozen, these syringes were taken out and pressed to collect the leaf sap from the thawed samples in Eppendorf tubes. Then 10 µL from each sample was taken and readings were obtained by vapor pressure osmometer (WESCOR 5520) in mmol/Kg and with the assistance of this formula, these values have been translated to (-MPA)

Osmotic potential = Osmolality (mmol) × 0.831 × 10^−5^ T (K) where T is temperature expressed in K

The membrane stability index (MSI) was found by following the procedure of Premchandra et al. (1990). For this, 100 mg of leaf discs were rinsed subsequently with tap and double-distilled water. Then, leaf discs were heated in 10 mL of double distilled water in the water bath for 30 min at 40 °C. EC meter was used to determine the initial electrical conductivity (C_1_) of all samples. The Second EC reading (C_2_) was recorded after placing the samples in a water bath for 10 min at 100 °C. The following formula was used for evaluating the membrane stability index

$MSI=(1-\frac{C1}{C2})\times 100$

#### 2.7.2 Biochemical parameters

A spectrophotometer method was used to determine the proline content (Bates et al., 1973). Proline content was determined by the following method. The plant extract was prepared in 4 mL of 3% sulfosalicylic acid (Sigma Chemical Co). Ninhydrin reagent was mixed in plant extract and absorbance was measured at 520 nm by a spectrophotometer. Soluble sugar was determined by following Dubois et al. (1956). Leaf material 0.5 g was grounded with pestle and mortar, then 10 mL of distilled water was mixed in it. Then this whole content was filtered after mixing well. The test tube was filled with 0.1 mL and 1 mL of 5% phenol was mixed in it and placed at room temperature for 1hr. Then 5 mL of H_2_SO_4_ was added to it and absorbance of the solution was recorded at 420 nm with a spectrophotometer. The standard curve of glucose suspension of the known volume was used to know the amount of sugar in the sample.

The protein content of the plant was estimated by following the procedure of Bradford (1976). The plant extract was made by grinding 0.2 g of leaf in 4 mL of phosphate buffer solution (pH 7) and then centrifuged it. The plant extract was mixed in distilled water with a volume of 0.5 mL each and then add 3 mL of coomassie bio rad dye in separate test tubes. The reaction mixture was kept for 5 min undisturbed and reading was recorded at 595 nm wavelength. For the determination of free amino acids, 1 mL of plant extract prepared for protein determination was taken, and then 1 mL of 10% pyridine and 1 mL of 2% ninhydrin solution was added. The absorbance of the mixture was recorded at 570 nm by spectrophotometer (Hitachi U-2000) (Hamilton and Slyke, 1943).

#### 2.7.3 Antioxidant enzyme assay

The extract of the enzyme was prepared by crushing 1 g of leaf in liquid nitrogen. The prepared extract was mixed in 10 mL of 50 mM phosphate buffer (pH 7.0) and 1 mM Ethylene Diamine Tetra Acetic Acid (EDTA) and 1% polyvinylpyrrolidone (PVP). At 13,000 rpm for 20 min at 4 °C, the entire content blend was centrifuged. The filtrate was utilized for enzyme analysis.

The degradation of H_2_O_2_ at 240 nm was reported to determine the catalase (CAT) content. The molar absorption coefficient of 40 mm^−1^ cm^−1^ for H_2_O_2_ was used to assess catalase activity (U mg protein^−1^) (Aebi, 1984). Peroxidase dismutase (POD) was found out by following the procedure of Rao et al. (1996). The total content is 10 μL crude enzyme extract, 20 μL 100 mM guaiacol, 10 μL 100 mM H_2_O_2_ and 160 μL 50 mM sodium acetate, respectively (pH 5.0). At 450 nm, absorbance was measured.

Superoxide dismutase (SOD) activity was measured by following the method of Beauchamp and Fridovich (1971). Whole content (3 mL) consisted of 13 mM methionine, 0.075 mM NBT, 0.1 mM EDTA, 0.002 mM riboflavin, and 0.1 mL of enzyme extract in 50 mM phosphate buffer (pH 7.8). The solution in the tube was kept in fluorescent light for 15 min. The reaction was stopped by turning off the lights. The absorbance was measured at 560 nm with a spectrophotometer. One unit of SOD activity was considered as the quantity of enzyme, which decreased the absorbance reading by 50% when compared to the control (lacking enzyme).

### 2.8 Statistical analysis

The software used for statistical analysis was Statistix 9.1. A two-way ANOVA with a factorial block design was carried out for all treatments. Each treatment had three replicates. Mean was compared by using Tukey multiple comparison post hoc tests.

## 3 Results

The present research was designed to observe the bio stimulating potential of biochar for remediating the crude oil contaminated soil and used it for maize plantation. Biochar acts as an absorbent for hydrocarbons and stimulates the microbial population in the soil to the breakdown of hydrocarbons. It was noted that biochar was not only effective in remediation but also neutralized the toxic nature of crude oil. The characteristics of biochar used have been mentioned in Table 1.

**Table 1:** Characterization of biochar.

pH	7.2
Moisture content	4.382%
Ash content	2.8%
Volatile matter	73.5%
Organic carbon	63.53%
N%	0.19
P%	0.16
K%	0.65
Na%	0.8
Mg	0.35

### 3.1 Soil analysis

Crude oil contamination adversely affected soil properties resulting in a reduction in pH of soil (3% and 5% at 10% and 15% contamination), EC (11% and 15.5% at 10% and 15% contamination), and soil moisture content (20.5% and 31.5% at 10% and 15% contamination). Results indicate a significant decline in available nutrients. The nitrogen, potassium, and phosphorus showed a maximum decrease by 22% and 28.35%, 6% and 11%, 7.5%, and 15% at 10% and 15% contamination, respectively as compared to the non-contaminated soil. On the other hand, organic carbon increased significantly by three and four times, at 10% and 15% oil level. Biochar improved soil properties both with and without oil stress. Biochar showed promising results by improving 20% and 15.65% in the moisture content of the soil at 10% and 15% oil contamination, 23% and 16% increase in nitrogen contents at 10% and 15% oil contamination, 10%-5% in phosphorous content and from 5% to 3% in potassium at 10% and 15% oil level, respectively (Table 2)

**Table 2:** Effect of biochar remediation on physiochemical properties of hydrocarbon contaminated soil.

**Treatments**	**pH**	**EC (dSm^−1^)**	**Soil Texture**	**Organic matter (g/kg)**	Available nutrients(%)		
					**N**	**P**	**K**
*T0*	6.99	0.72	Sandy clay loam	7.72	0.47	2	0.55
*T1*	6.75	0.65	Sandy clay loam	6.54	0.29	1.82	Traces
T2	6.44	0.56	Sandy clay loam	5.97	0.24	1.70	Traces
*T3*	7.01	0.79	Sandy clay loam	8.92	0.66	2.25	0.830
*T4*	6.80	0.69	Sandy clay loam	6.70	0.49	1.90	0.63
*T5*	6.5	0.60	Sandy clay loam	6.30	0.30	1.79	0.56

Where, T0 = Control soil T1 = 10% contaminated soil with crude oil, T2 = 15% contaminated soil with crude oil, T3 = Biochar + Control Soil, T 4 = Biochar + 10% contaminated soil with crude oil, T5 = Biochar + 15% contaminated soil with crude oil.

### 3.2 Soil enzymatic activity

**Fig. 1:**
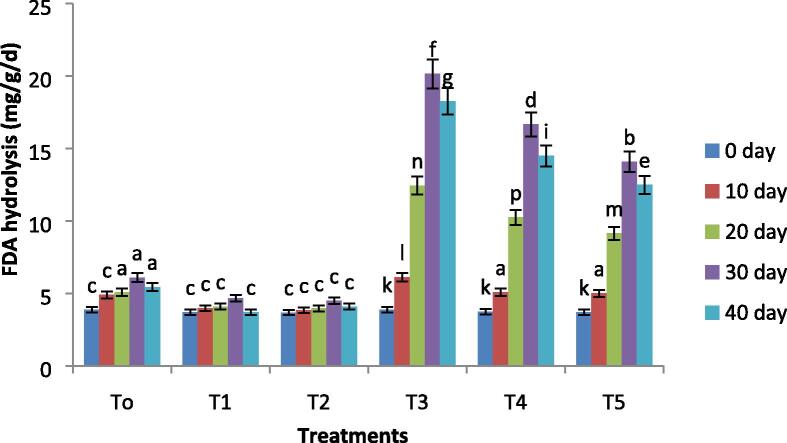
Variations in the fluorescein diacetate (FDA) hydrolysis of all treatments with remediation time. Where, T0 = Control soil T1 = 10% contaminated soil with crude oil, T2 = 15% contaminated soil with crude oil, T3 = Biochar + Control Soil, T 4 = Biochar + 10% contaminated soil with crude oil, T5 = Biochar + 15% contaminated soil with crude oil.

**Fig. 2:**
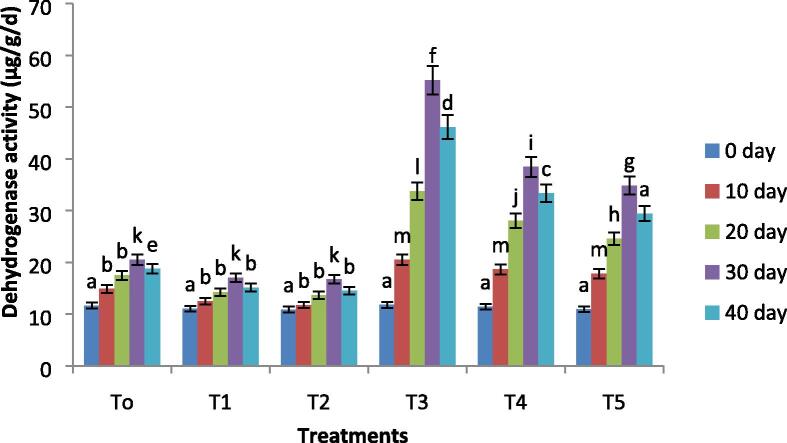
Variations in the dehydrogenase activity of all treatments with remediation time. Where, T0 = Control soil T1 = 10% contaminated soil with crude oil, T2 = 15% contaminated soil with crude oil , T3 = Biochar + Control Soil, T 4 = Biochar + 10% contaminated soil with crude oil, T5 = Biochar + 15% contaminated soil with crude oil.

Important enzyme activity including dehydrogenase activity and hydrolysis of fluorescein diacetate (FDA) was assessed after every 10 days and the results are shown in Fig. 1 and Fig. 2. The activities of both enzymes initially decreased and gradually increased significantly at 30 days of the experiment (p≤0.05). While no change was observed in both enzymatic activity in the control condition. The biochar incubation considerably increased enzyme action in both the presence and absence of oil contamination. The activity of FDA hydrolysis was 38.5% and 25.04% increased with biochar treatment soil at 10% and 15% level of oil contamination as compared to the initial day of the experiment. This was 13 and 10 times greater in biochar treated soil with respect to the initial days of the experiment. However a percent increase (Fig. 1). Biochar incubated soil had shown a 55.6% and 41.2% increase in activity of dehydrogenase at 10% and 15% level of oil contamination as compared to the initial days of the experiment (Fig. 2). This was a 27 and 24 fold increase in biochar-treated soil as compared to the initial days of the experiment.

### 3.3 Petroleum degrading bacterial count and respiration rate

**Fig. 3:**
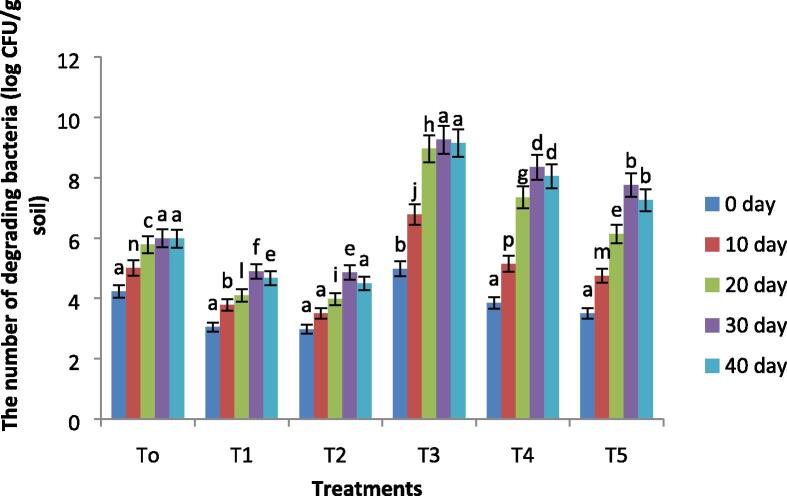
Variations in the microbial diversity of all treatments with remediation time. Where, T0 = Control soil T1 = 10% contaminated soil with crude oil, T2 = 15% contaminated soil with crude oil , T3 = Biochar + Control Soil, T 4 = Biochar + 10% contaminated soil with crude oil, T5 = Biochar + 15% contaminated soil with crude oil.

**Fig. 4:**
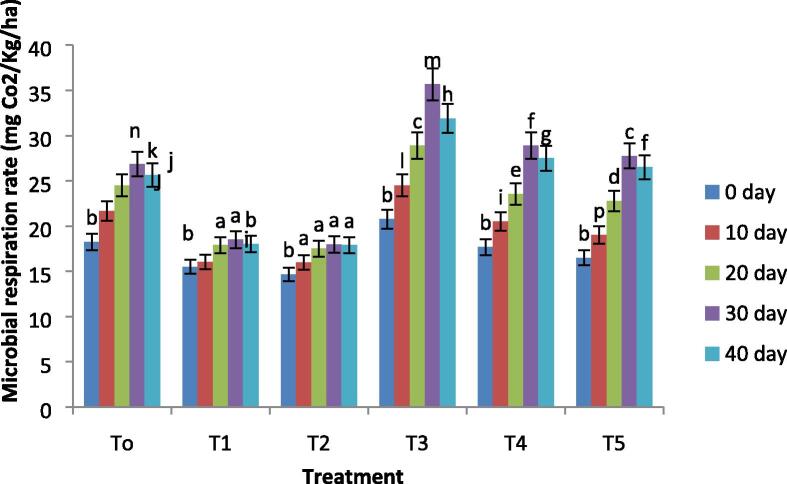
Variations in the microbial respiration rate of all treatments with remediation time. Where, T0 = Control soil T1 = 10% contaminated soil with crude oil, T2 = 15% contaminated soil with crude oil, T3 = Biochar + Control Soil, T 4 = Biochar + 10% contaminated soil with crude oil, T5 = Biochar + 15% contaminated soil with crude oil.

The production of CO_2_ is considered to be an integral attribute for the development and propagation of petroleum degrading bacteria. Data regarding microbial respiration and the bacterial count had shown a significant increase with biochar incubation in comparison to control one both in the presence and absence of oil (p≤0.05). The gradual increase in microbial respiration and bacterial diversity was recorded at 30 days of the experiment. At 40 days of the experiment, a slight decrease in the microbial activities was noted (Fig. 3, Fig. 4). Biochar amendment in oil-contaminated soil had shown a 41% and 35% increase in the bacterial count at 10% and 15% level of oil as compared to untreated soil, respectively. A similar increase in microbial respiration was observed in soil incubated with biochar with respect to control one. Biochar incubation has resulted in a 10 and 8 folds increase in microbial respiration at 10% and 15% oil-contaminated as compared to a respective control

### 3.4 Hydrocarbons degradation

**Fig. 5:**
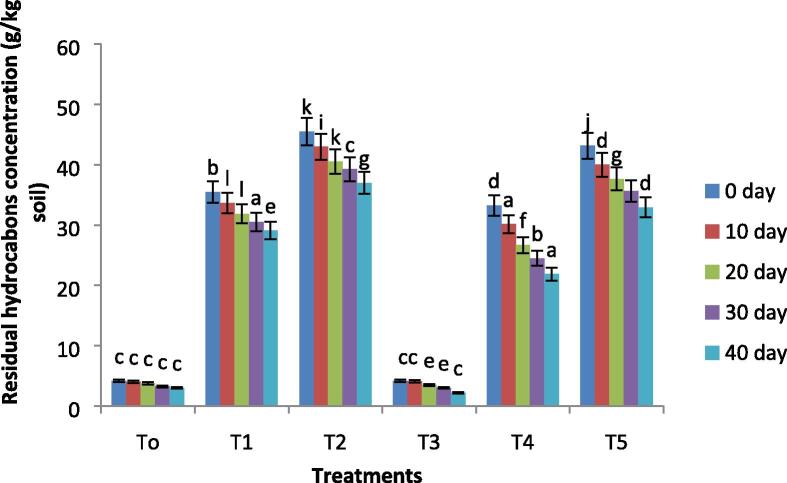
The residual hydrocarbons concentration of all treatments with remediation time. Where, T0 = Control soil T1 = 10% contaminated soil with crude oil, T2 = 15% contaminated soil with crude oil, T3 = Biochar + Control Soil, T 4 = Biochar + 10% contaminated soil with crude oil, T5 = Biochar + 15% contaminated soil with crude oil.

**Fig. 6:**
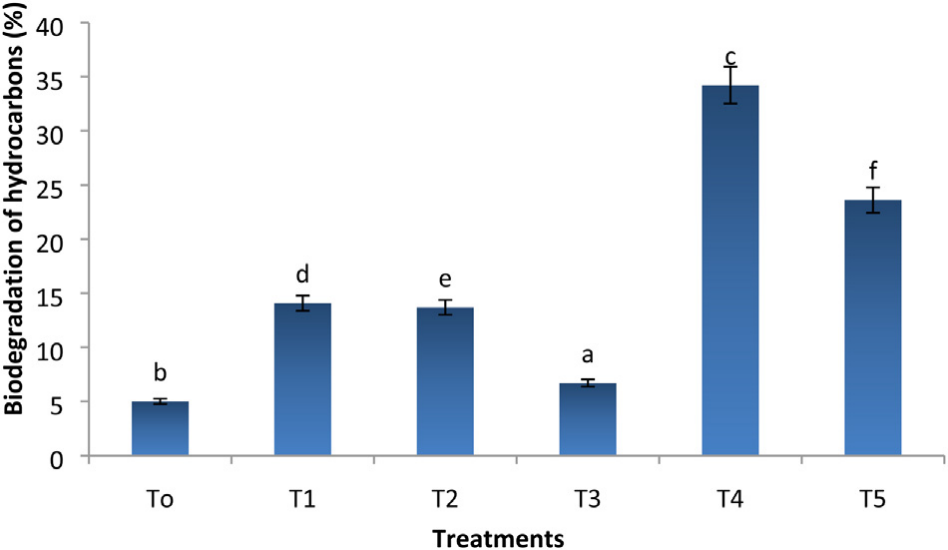
Biodegradation of hydrocarbons (%) of all treatments. Where, T0 = Control soil T1 = 10% contaminated soil with crude oil, T2 = 15% contaminated soil with crude oil, T3 = Biochar + Control Soil, T 4 = Biochar + 15% contaminated soil with crude oil, T5 = Biochar + 15% contaminated soil with crude oil.

Total petroleum hydrocarbons (TPHs) were found after 40 days in soil samples obtained from all treatments. As illustrated in Fig. 5, the TPHs concentration significantly decreased in biochar treated soil under oil contamination (p≤0.05). Biochar incubated soil had shown 11 folds decrease in TPHs concentration after 40 days of the experiment as compared to the initial days of the experiment. A significant increase in the biodegradation efficiency of biochar has been noted as compared to untreated samples. Biochar amendment resulted in 34.2% and 23.6% hydrocarbon degradation at 10% and 15% level of oil as compared to control one, respectively (Fig. 6).

Gas Chromatography-Mass Spectrometry (GC-MS) analysis of soil samples was carried out for the determination of compounds generated after the 40 days trial. The results suggested that crude oil was degraded in soil by biochar treatment after 40 days trial. The mass spectrometer identifies the compounds using NIST library. We found a range of n-alkanes (C_5_-C_18_) presented in the Table 8. The total ion chromatograms were created from the data, which indicated the conversion of crude oil to be linked with the production of metabolites and provided useful knowledge about the method of biodegradation. Soil treated with biochar had shown a high proportion of alkanes (C_9_-C_12_) such as Benzene, 1,2,4-trimethyl, Decane, 4-methyl, Dodecane which indicated the degradation of aromatic hydrocarbons while the higher content of alkanes (C_10_-C_18_) in oil-contaminated soil indicates the recalcitrant hydrocarbons not degraded after 40 days trial (Table 8).

### 3.5 Plant biomass

Plant fresh biomass (Table 3) showed a significant reduction (32%, 46%) plant dry biomass (17.53%, 21.36%) at 10% and 15% level of oil and leaf area (11.11% and 125% in 10% and 15% contaminated soil). Soil amendment with biochar helped to mitigate the effect of crude oil and increased by 25.5% and 18.2% fresh biomass and 14.61% and 8.7% dry biomass at 10% and 15% level of oil contamination as compared to control plant, respectively. A percent increase of 9.16% and 8% was observed by the incubation of biochar at 10% and 15% level of oil-polluted soil (p≤0.05).

**Table 3:** Leaf area, Fresh and dry biomass of maize growing in hydrocarbon contaminated and biochar remediated soil.

**Treatments**	**Fresh biomass (g)**	**Dry biomass (g)**	Leaf area (cm^2^)
To	12.5 ± 0.62f	3.65 ± 0.5b	135 ± 0.01b
T1	8.5 ± 0.26e	3.01 ± 0.62a	120 ± 0.5d
T2	6.75 ± 0.01a	2.87 ± 0.24d	118 ± 0.89e
T3	15.5 ± 0.05b	4.89 ± 0.01e	151 ± 0.49a
T4	10.67 ± 0.49d	3.45 ± 0.05c	131 ± 0.56c
T5	7.98 ± 0.81e	3.12 ± 0.26f	127 ± 0.41f

The mean and standard deviation (n = 3) are displayed in this data. Significant variations are seen in different letters (p < 0.05). Detail of treatments as in Table 1.

### 3.6 Physiological parameters

Crude oil's impact on photosynthetic pigments was more pronounced as compared to other parameters. Total chlorophyll content in maize plants decreased persistently due to continuous increment in the level of oil in soil (Table 4). Oil contamination resulted in a 29.9% and 40.3% decrease in total chlorophyll content of plants at 10% and 15% oil level with respect to control plant respectively (p≤0.05). Incubation of soil with biochar improved the total chlorophyll content by 15.49% in the absence of oil in contrast to the untreated one. A considerable improvement of 11.7% and 8.02% was observed in the total chlorophyll content of plants at 10% and 15% level of oil as compared to the control plant, respectively. A significant decrease of 28.2% and 38.3% in chlorophyll *a* of plants was observed at 10% and 15% level of oil as compared to control one, respectively. Biochar incubation resulted in a 24.6% increase in chlorophyll content in the absence of oil, 16.4%, and 10% increase in chlorophyll at 10% and 15% level of oil as compared to the control one. Similarly, oil contamination resulted in a considerable decrease of 26.5% and 29.5% in the content of chlorophyll *b* of the plant in comparison to uncontaminated plant, respectively. The addition of biochar improved the 19.5% chlorophyll *b* of the plant as compared to the control one in the absence of oil. While, 11.96% and 9.6% increase was recorded in biochar treated plants at 10% and 15% level of oil as compared to control plant, respectively.

**Table 4:** Chlorophyll *a*, b and total chlorophyll of maize growing in hydrocarbon contaminated and biochar remediated soil.

**Treatments**	**Chlorophyll *a* (mg/g fresh weight)**	**Chlorophyll *b* (mg/g fresh weight)**	Total Chlorophyll (mg/g fresh weight)
To	7.3 ± 0.62f	4.1 ± 0.05b	12.13 ± 0.05e
T1	5.24 ± 0.89b	3.01 ± 0.41f	8.5 ± 0.26a
T2	4.5 ± 0.65d	2.89 ± 0.26a	7.23 ± 0.62c
T3	9.1 ± 0.5e	4.9 ± 0.49e	14.01 ± 0.45d
T4	6.1 ± 0.01c	3.37 ± 0.89c	9.45 ± 0.5b
T5	4.95 ± 0.02a	3.2 ± 0.91d	7.81 ± 0.89f

The mean and standard deviation (n = 3) are displayed in this data. Significant variations are seen in different letters (p < 0.05). Detail of treatments as in Table 2.

Leaf osmotic potential was significantly decreased 26.97% and 37.67% in maize plants at 10% and 15% level of oil contamination when compared to control one (p≤0.05). Biochar treatment tended to reduce the oil effect resulting in a 21.6% and 16.5% increase in osmotic potential of plants, in contrast, to control plants respectively. However, the leaf water potential of oil-exposed plants decreased at 10% and 15% level of oil. Biochar treatment also enhanced the osmotic potential in the presence of oil as compared to control plants (Table 5).

**Table 5:** Water potential, osmotic potential and membrane stability index (MSI) of maize growing in hydrocarbon contaminated and biochar remediated soil.

**Treatments**	**Water potential (-MPa)**	**Osmotic potential (-MPa)**	MSI
To	0.1 ± 0.05a	2.15 ± 0.5b	83.45 ± 0.41a
T1	0.77 ± 0.01d	2.73 ± 0.25d	55.67 ± 0.78d
T2	0.96 ± 0.26e	2.96 ± 0.49f	46.25 ± 0.68e
T3	0.1 ± 0.61f	2.21 ± 0.64e	97.86 ± 0.52c
T4	0.74 ± 0.49c	3.32 ± 0.89c	62.75 ± 0.65f
T5	0.91 ± 0.5b	3.45 ± 0.21a	51.45 ± 0.89b

The mean and standard deviation (n = 3) are displayed in this data. Significant variations are seen in different letters (p < 0.05). Detail of treatments as in Table 2.

A significant reduction of 33.28% and 44.57% was observed in membrane stability index of maize plant at 10% and 15% contamination crude oil contamination as compared to control one (p≤0.05). Biochar incubation had shown an increase in membrane stability index of the plant at both presence and absence of oil stress. A percent increase of 17.26% was recorded in the membrane stability index of the maize plant in the absence of oil, while an increase of 12.71% and 11.24% was observed at 10% and 15% level of oil as compared to control one (Table 5).

### 3.7 Effect on compatible solutes

A considerable amount of osmolytes was accumulated in oil-stressed plants (p≤0.05). In contrast to the control plant, a 13.9% and 23.2% increase in proline content was observed in plants at 10% and 15% level of oil contamination (Table. 6). Biochar incubation allowed the plant to maintain a high level of proline up to 26% and 29% at 10% and 15% level of oil contamination as compared to control plants respectively. A similar increase of 18.5% and 22.7% insoluble sugar content was observed at 10% and 15% level of oil contamination, in contrast, to control plants respectively. The effect of biochar incubation remained significant under oil stress. A 23.9% and 30.4% increase in soluble sugar content was observed at 10% and 15% level of oil contamination as compared to the control plant respectively. Due to the rising concentration of oil in the soil, total soluble proteins in the leaves of maize plants have decreased significantly. Biochar incubation had shown a significant increase both in the absence and presence of oil stress. Crude oil contamination elevated the level of free amino acids of plants. A significant increase of 15% and 29.5% in free amino acids was observed at 10% and 15% level of oil contamination, in contrast, to control plants respectively. Treatment of biochar resulted in a 14.6% and 16.15% increase in the amino acid content of plants at 10% and 15% level of oil for control plants respectively (Table 6).

**Table 6:** Proline, total amino acid, total soluble sugar and total protein content of maize growing in hydrocarbon contaminated and biochar remediated soil.

	**Proline (µg g^−1^ FW)**	**Total Amino Acid (µg g^−1^ FW)**	**Total Soluble Sugar (µg g^−1^ FW)**	Total Protein Contents (µg g^−1^ FW)
Treatments				
To	2.15 ± 0.01b	9.08 ± 0.5b	4.09 ± 0.02a	1.16 ± 0.49e
T1	2.5 ± 0.04a	10.45 ± 0.89d	4.85 ± 0.43d	1.09 ± 0.81d
T2	2.65 ± 0.23c	11.76 ± 0.45f	5.02 ± 0.01e	1.06 ± 0.26b
T3	2.34 ± 0.63f	9.65 ± 0.5c	4.45 ± 0.2b	1.20 ± 0.41c
T4	3.15 ± 0.26e	11.98 ± 0.23a	6.01 ± 0.62f	1.12 ± 0.32f
T5	3.42 ± 0.41d	11.95 ± 0.01e	6.55 ± 0.26c	1.11 ± 0.21a

The mean and standard deviation (n = 3) are displayed in this data. Significant variations are seen in different letters (p < 0.05). Detail of treatments as in Table 2.

### 3.8 Plant antioxidants

A notable rise in antioxidants was observed in crude oil contaminated soil (Table 7). An increase in superoxide dismutase activity (SOD) was noted by 21.4% and 32.14% in maize plants grown under 10% and 15% contamination, respectively (p≤0.05). Biochar increased SOD by 16.17% and 17.56%, at 10% and 15% contamination, respectively. A major improvement in catalase activity by 18.2% and 21.6% was observed in plants grown under 10% and 15% contamination, respectively. The increase in catalase by biochar was 11.38% and 9.8% at 10% and 15% crude oil contamination when compared to the control plant respectively. Similarly, plants showed pronounced peroxidase activity by 17.02% and 21.2% at 10% and 15% oil contamination, respectively. Biochar treatment showed a significant increase of 12.7% and 10.5% in activity of peroxidase dismutase at 10% and 15% crude oil contamination as compared to control plants, respectively.

**Table 7:** Catalase, peroxidase and superoxide dismutase content of maize growing in hydrocarbon contaminated and biochar remediated soil.

	**Superoxid Dismutase (EU mg^−1^ Protein)**	**Catalase (EU mg^−1^ Protein)**	Peroxidase (EU mg^−1^ Protein)
Treatments			
To	1.12 ± 0.05d	2.08 ± 0.62a	141 ± 0.05e
T1	1.36 ± 0.26e	2.46 ± 0.5c	165 ± 0.5d
T2	1.48 ± 0.49d	2.53 ± 0.02e	171 ± 0.41a
T3	1.28 ± 0.81a	2.35 ± 0.42b	155 ± 0.61f
T4	1.58 ± 0.01b	2.74 ± 0.89f	186 ± 0.81b
T5	1.74 ± 0.3c	2.78 ± 0.23d	189 ± 0.08c

The mean and standard deviation (n = 3) are displayed in this data. Significant variations are seen in different letters (p < 0.05). Detail of treatments as in Table 2.

**Table 8:** The crude oil degrading products generated after biodegradation by biochar.

**Treatments**	**Retention Time**	**Peak Area (%)**	**Compounds**	**Molecular Formula**	M.W (g/mol)
T0	12.5	21485 × 10^3^	Tridecane	C_13_H_28_	184.37
	14.67	4506 × 10^3^	Dodecane	C_12_H_26_	170.33
	15.25	3876 × 10^3^	Undecane	C_11_H_24_	156.31
T1	17.1	1309 × 10^3^	1,2,3,5-Tetramethylbenzene	C_10_H_14_	134.22
	14.65	5891 × 10^3^	Pentadecane	C_15_H_32_	212.42
	11.35	11245 × 10^3^	Hexadecane	C_16_H_34_	226.41
	12.45	26811 × 10^3^	Benzene, 1,1′-ethylidenebis-	C14H12Br2	340.05
	15.6	36512 × 10^3^	Octadecane	C_18_H_38_	254.5
T2	16.5	11421 × 10^3^	n-Hexadeconic acid	C_16_H_32_O_2_	256
	14.65	31141 × 10^3^	7,9 Di-*tert*-butyl-1-oxaspiro(4,5)	C_17_H_24_O_3_	276
	10.25	13412 × 10^3^	Pyrene	C_16_H_10_	202
	15.67	41123 × 10^3^	Octadecane	C_18_H_38_	254.5
	16.24	5467 × 10^3^	Flouranthene	C_16_H_10_	202
	17.5	12871 × 10^3^	2-Bromotetradecane	C_14_H_29_Br	276
T3	18.1	45621 × 10^3^	Cyclopentane	C_5_H_10_	70.1
	13.65	34611 × 10^3^	Benzaldehyde	C₇H₆O	106.12
T4	16.87	23141 × 10^3^	Benzene, 1,2,4-trimethyl-	C_9_H_12_	120.19
	19.1	11261 × 10^3^	Decane, 4-methyl-	C_11_H_24_	156.31
	11.65	10231 × 10^3^	Dodecane	C_12_H_26_	170.33
T5	13.45	4231 × 10^3^	Tridecane	C_13_H_28_	184.37
	12.65	11212 × 10^3^	1-ethyl-2-methylbenzene	C_9_H_12_	120.19
	16.75	46812 × 10^3^	Decane, 4-methyl-	C_11_H_24_	156.31

### 3.9 Heatmap response of Pearson’s correlation coefficient (r)

**Fig. 7:**
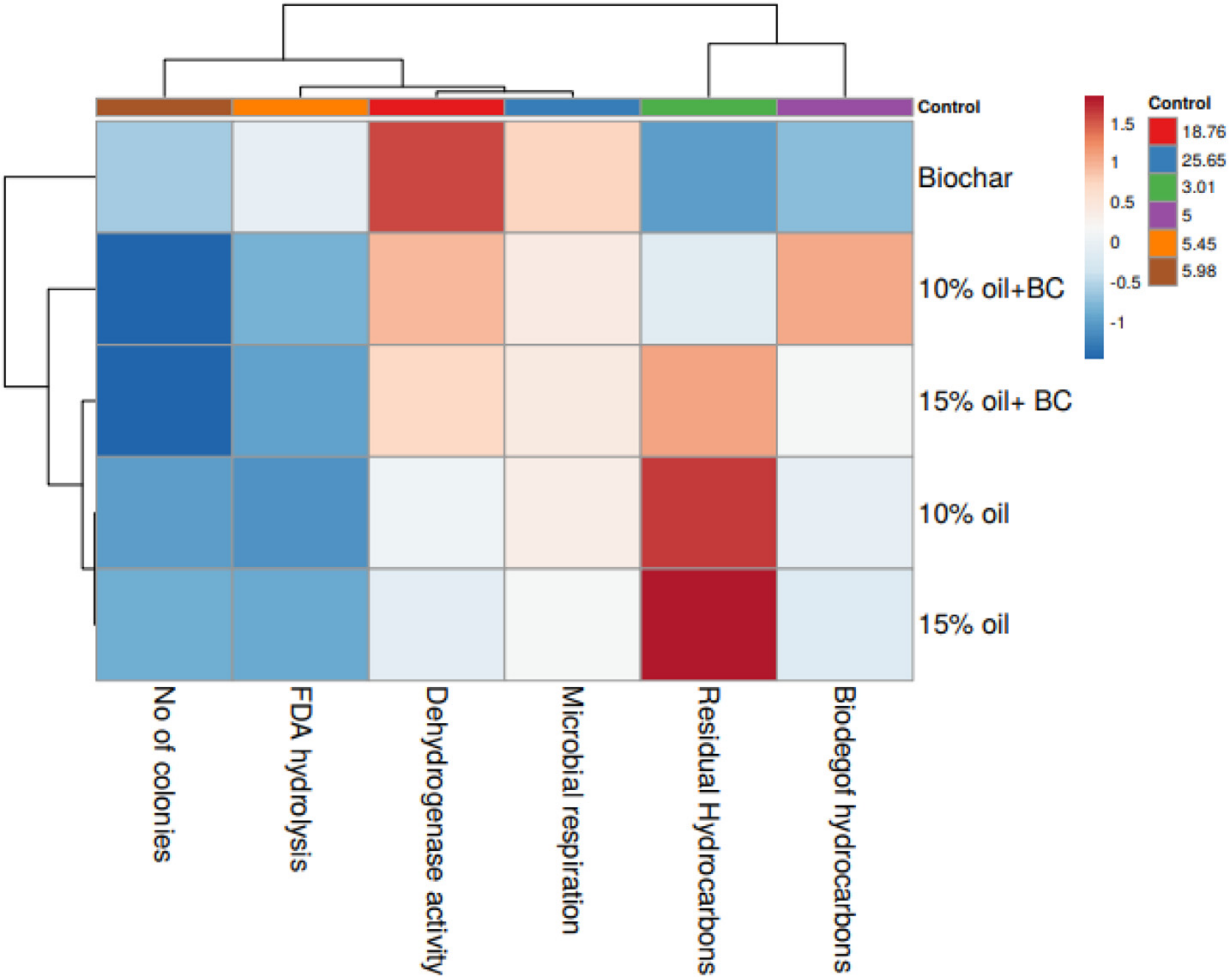
A heat map showing the correlation between different parameters and treatments.

For heat map analysis the data of soil under crude oil stress were characterized as soil enzymatic activity, soil microbial diversity, microbial respiration rate, residual hydrocarbon content, and biodegradation rate and each attribute gives positive correlations (Fig. 7). A comparative study of the factors associated with hydrocarbons degradation justified that degradation of hydrocarbons in oil-contaminated soil had a positive correlation with soil enzymatic activities, number of oil-degrading microbes, rate of microbial respiration, and concentration of residual hydrocarbons. These results indicate the biodegradation efficiency of biochar for remediation of crude oil-contaminated soil.

## 4 Discussion

Crude oil contamination is one of the major environmental issues now a day. Although many remediation techniques for crude oil contamination have been proposed, sill development of an eco-friendly method is highly recommended. Biostimulation is currently being used methods for the bioremediation of crude oil-contaminated soil. So, the present research was designed to observe the biostimulation potential of biochar for remediation of crude oil-contaminated soil. We found that this method is not only beneficial to remediate the soil but also capable to overcome the toxic nature of crude oil.

Biochar improved soil characteristics not only under control conditions (without any contamination) but also under oil contamination. Treatment of soil with biochar showed the most promising results in the degradation of crude oil. An inverse relationship exists between the rate of hydrocarbon degradation and the level of oil contamination as hydrocarbon degradation decreases with an increase in the oil contamination. Biochar degraded the crude oil contaminants in more members of low molecular weight compounds at both contamination levels (10% and 15%). Biochar has shown the potential of oil degradation and promotes microbial degradation. Galitskaya et al. (2016) also reported that organic compounds in crude oil could be metabolized by oil-degrading microbes which are stimulated by soil amendment. This can be justified by the fact that components of biochar act as a substrate for microbes or making the soil more suitable for the growth of microbes. Bioremediation of crude oil polluted soil by stimulating the activity of bacteria by the addition of poultry manure was also documented. Biochar can enhance the absorption of organic contaminants. The biochar increases the soil nutrient content for microbes containing organic carbons and hydrophobic organic compounds (HOCs), like polyaromatic hydrocarbons (PAHs), which bind more closely (Kong et al., 2018).

The enzyme actions, such as fluorescein diacetate (FDA) hydrolysis and dehydrogenase, were determined every 10 days and the effects are given in Fig. 1, Fig. 2. Many researchers have acknowledged that the enzyme actions of soil are interrelated with the fertility of soil and biomass of microbes (Dong et al., 2014), which is considered as the best sign of quality and health of the soil. The activities of both enzymes progressively improved at the initial stage and then reduced to some extent at the end of the experiment, while the change in the control treatment was not noticeable. These variations have shown that the incubation of biochar can act as a carbon source for native microbes and stimulate the growth and behavior of microbes in the soil. Biochar act as a source of carbon for microbes to stimulate enzymes and biodegradation of pollutants (Jiang et al., 2016). Enzyme actions reduce to some extent at 40 days and the promising reason for such decrease is an incomplete decomposition of biochar and presence of residual hydrocarbons inhibiting the microbial activity, resulting in exfoliation and death as well.

Microbial respiration is considered to be a significant soil biological index that can reveal the use of total petroleum hydrocarbons (TPHs) by microbes. Previous reports have explained that the emission of CO_2_ is a significant attribute for microbe development, metabolism, and reproduction in oil-polluted soils (Wang et al., 2019). As presented in Fig. 3, Fig. 4. in the initial stage, the rate of respiration and diversity of hydrocarbons degrading bacteria progressively improved, and reduces after 30 days of remediation, parallel to enzymatic activities during the remediation process. The possible justification is the abundance of nutrients and favorable conditions at the early stage of the experiment. At 40 days, a living environment for microbes becomes unfavorable due to a reduction in redox potential and nutrients like C, N, and P. However, biochar incubation can considerably stimulate number and microbial activity in oil-polluted soil (Sarma et al., 2019).

Hydrocarbons polluted water and soil are considered to be hazardous for the ecosystem. Reduction in maize growth was observed due to oil contamination, such findings are in accordance with earlier results in which reduction in shoot growth has been recorded in plants growing in oil-contaminated soil. (Tomar et al., 2016). Results of current research show that the fresh and dry biomass of plants was reduced in oil-contaminated soil (Table. 3). More reduction was encountered at a 15% level of oil as compared to a 10% level of oil. Plants growing in oil-contaminated soil are documented to have a reduction in growth due to the accumulative impact of toxicity of hydrocarbons and inadequate aeration because of blockage of soil pores with crude oil (Pernar et al., 2006). Soil with a high level of oil has led to an increase in growth inhibition because of difficulty in the water and ion absorption. The possible justification of this fact that absorption of toxic contaminants by plants can change the structure and function of the plasma membrane. A similar decline in shoot length of maize plant on exposure to oil was recorded by Athar et al. (2016). Various researches have explained the effect of remediation techniques in the improvement of plant growth (Shahid et al., 2017). A significant increase in plant fresh and dry biomass was recorded in biochar treated plants at 10% and 15% level of oil contamination (Table. 3). Our findings are parallel with the results of Laird (2010), who also observed the increase in fresh and dry biomass due to incubation of biochar. Biochar addition resulted in a reduction in loss of soil nutrients as biochar added nutrients in the soil.

Photosynthesis is central to the growth of the plant. From the result of the present study, a substantial decrease in the content of chlorophyll *a*, b, and total chlorophyll content was noted in plants growing in oil-polluted soil with respect to uncontaminated soil conditions (Table. 4). Baruah et al (2014) have been documented a similar reduction in chlorophyll content. The addition of biochar increased the chlorophyll content of plants in oil-contaminated soil. The increase is due to enhanced uptake of nutrients and reduction in oil uptake (Mosa et al., 2016). Such reduction in total chlorophyll content is similar to the results of already reported literature in which reduction in photosynthetic pigments or chlorosis of leaves due to oil contamination has been explained (Ali et al., 2017). The pressure potential of the plant can be maintained by decreasing the osmotic potential. (Borgo et al., 2015). While the addition of biochar leads to improve soil nutrients and water use efficiency and thus crop yield (Haider, 2015).

Accumulation of compatible solutes is the common response of stress-exposed plants. Oil contamination leads to a considerable improvement in the production of proline, soluble sugar, and free amino acid content as compared to control plants (Table 6). While a considerable reduction in the amount of protein in oil-impacted plants was encountered with respect to the control plant. Proline is considered to be an osmotic stress protectant in response to tolerance to environmental stress (Bashir, 2014). In stress conditions, proline plays an important role in membrane stabilization and other cellular structure by the synthesis of reactive oxygen species. It also maintains the pH and turgor of the cell (Nazarli et al., 2011). Similar findings were lined with Wang et al (2014). Amino acids act as osmoregulators for plants in stress (Haider, 2015). Elevated sugar level under stress conditions, helps to maintain physiological roles such as photosynthesis, nutrient mobilization, and exports while less sugar level stimulates the storage of carbohydrates and senescence (Sami et al., 2016). Biochar incubation increased the soluble sugar of stress-exposed plants as compared to control one.

Antioxidants protect the cell from any damage resulted from cytotoxic O_2_, and stopover its conversion to H_2_O_2_ and O_2_ in all the organelles. Biochar treatment showed a significant increase of 12.7% and 10.5% in treated plants at 10% and 15% crude oil contamination (Table 7). The processes of remediation may stimulate the antioxidants by stimulating the uptake of nitrogen and phosphorous, which interact with carbohydrates as non-enzymatic antioxidants (Liu et al., 2010a, Liu et al., 2010b).

Correlation analysis indicates that degradation of hydrocarbons in oil-contaminated soil is positively correlated with the growth of maize plant by maintaining plant defense response including osmolytes and antioxidants enzymes production. This study establishes a relationship between the bio stimulating potential of biochar for hydrocarbons degradation with improved soil properties and stimulatory effect on plant growth.

## 5 Conclusion and future perspectives

Biostimulation is considered to be an efficient method for remediation of crude oil-contaminated soil. Biochar incubation in crude oil contaminated soil stimulates hydrocarbon degradation by accelerating the activities of microbes. The results of the present research have proven the bio stimulating effect of biochar for bioremediation of oil-contaminated soil and significant growth potential for maize plants. Biochar enhanced the soil microbial and enzymatic activities to degrade the hydrocarbons. It also enhances the morphological, physiological, and biochemical parameters of the plant. So, biochar can be used as a bio stimulating tool for remediation of oil-contaminated soil and hence can be used at a large scale on soils where crude oil contamination is a major problem, particularly for the agricultural sector.

## Declaration of Competing Interest

The authors declare that they have no known competing financial interests or personal relationships that could have appeared to influence the work reported in this paper.
